# A Biodegradable Magnetic Nanocomposite as a Superabsorbent for the Simultaneous Removal of Selected Fluoroquinolones from Environmental Water Matrices: Isotherm, Kinetics, Thermodynamic Studies and Cost Analysis

**DOI:** 10.3390/polym12051102

**Published:** 2020-05-12

**Authors:** Geaneth Pertunia Mashile, Kgokgobi Mogolodi Dimpe, Philiswa Nosizo Nomngongo

**Affiliations:** 1 Department of Chemical Sciences, University of Johannesburg, Doornfontein Campus, P.O. Box 17011, Doornfontein 2028, South Africa; petmashile2009@hotmail.com (G.P.M.); mdimpe@uj.ac.za (K.M.D.); 2 DSI/NRF SARChI Chair: Nanotechnology for Water, University of Johannesburg, P.O. Box 17011, Doornfontein 2028, South Africa; 3 DSI/Mintek Nanotechnology Innovation Centre, University of Johannesburg, P.O. Box 17011, Doornfontein 2028, South Africa

**Keywords:** fluoroquinolones, ultrasound radiation, mesoporous carbon, desirability function, thermodynamics, wastewater, cost analysis

## Abstract

The application of a magnetic mesoporous carbon/β-cyclodextrin–chitosan (MMPC/Cyc-Chit) nanocomposite for the adsorptive removal of danofloxacin (DANO), enrofloxacin (ENRO) and levofloxacin (LEVO) from aqueous and environmental samples is reported in this study. The morphology and surface characteristics of the magnetic nanocomposite were investigated by X-ray diffraction (XRD), Brunauer–Emmett–Teller (BET) adsorption–desorption and Fourier transform infrared spectroscopy (FTIR). The N_2_ adsorption–desorption results revealed that the prepared nanocomposite was mesoporous and the BET surface area was 1435 m^2^ g^−1^. The equilibrium data for adsorption isotherms were analyzed using two and three isotherm parameters. Based on the correlation coefficients (R^2^), the Langmuir and Sips isotherm described the data better than others. The maximum monolayer adsorption capacities of MMPC/Cyc-Chit nanocomposite for DANO, ENRO and LEVO were 130, 195 and 165 mg g^−1^, respectively. Adsorption thermodynamic studies performed proved that the adsorption process was endothermic and was dominated by chemisorption.

## 1. Introduction

The presence of pharmaceuticals in aquatic environments has become a subject of interest for environmental chemists [1]. Their wide distribution owes itself to the growing need for treatments and cures for human and animals diseases [2]. They are introduced into the aquatic environments through effluents of urban wastewater treatment plants (WWTPs) [3]. This is a result of their extensive use and their ineffective removal processes by wastewater transport and treatment [4]. Among various pharmaceuticals, antibiotics residues have proved to be the most commonly detected in the aquatic environment for both surface and ground waters [5]. Although they may occur in fairly low concentrations in environmental waters, their different modes of action and particular chemical and physical characteristics may pose a risk to the aquatic system [6]. Thus, there is a need to monitor and evaluate their persistent presence, which even at a low level can further increase antibiotic resistance [7]. The focus of this work is mainly on fluoroquinolones which are an important emerging group of synthetic antibacterials [8]. They have been used extensively for both human and veterinary medicine due to their effectiveness against both gram-positive and negative bacteria for the treatment of bacterial infections [2]. Moreover, different antibiotics have different half-lives; therefore, others may be more persistent in the environment which may result in increased levels of contamination to the environment [9].

Studies have shown that they are introduced to environmental bodies by either direct or indirect pathways [4,10,11]. Furthermore, they have been found to occur in surface waters at concentrations ranging from ng L^−1^ to µg L^−1^ [10,12]; ng L^−1^ to mg L^−1^ in groundwater [13]; and mg L^−1^ in soil [14]. Since they are continuously introduced into the environment they have been identified as pseudo-persistent organic pollutants [11]. The greatest challenge is the removal of antibiotics from wastewater before discharge into the environment due to the high costs associated with it [9]. Techniques such as advanced oxidation processes (AOPs), multi-treatment processes, separation processes and biological processes have been applied in the removal of antibiotics from wastewater [15]. However, they prove to be very expensive and require high maintenance for the complete removal of compounds, including antibiotics, at a larger scale [16].

Adsorption processes are of significant interest in removal applications of organic compounds such as antibiotics due to their simplicity in design [17], flexibility, cost and friendliness towards potential the toxicity of biological base processes [18]. The adsorption is a technique based on the removal of contaminants from a matrix onto an adsorbent surface [19]. The effectiveness of the technique is highly dependent on the adsorbate properties, adsorbent type and composition of matrix analyzed [20]. To date, various adsorptive material has been used, such as zeolites [21], graphene oxide (GO) [22,23], activated carbon (AC) [24,25,26,27,28], metal-organic frameworks (MOFs) [29], carbon nanotubes (CNT) [30] and clay [31], amongst others for adsorption removal of pharmaceuticals [32]. However, for antibiotic removal, CNTs, ACs, mesoporous clay material, exchange resins and bentonite are the most widely reported adsorbents [9]. Despite their widespread use, these sorbents also present some limitations, such as inefficient extraction, low antibiotic adsorption properties and costliness (high generation costs) [9]. Mesoporous carbon from carbon-based material, on the other hand, can serve as an artery for adsorbates and also contribute greatly towards adsorption [33,34]. It can boast advantageous features, such as a large surface area; a high adsorption capacity; a large and ordered pore size and structure; and chemical and mechanical stability [28,33,34,35,36,37,38,39]. Furthermore, mesoporous carbon can be made from cheaper materials, such as starch and waste biomass [28,33,37,38,39]. In addition, the incorporation of magnetic nanoparticles to mesoporous carbon facilitates ease during separation, and functionalizing the material enables for reduction of its hydrophobic nature [38,39].

Furthermore, the natural polymers such as chitosan and beta-cyclodextrin have gained prominence in recent years due to their advantageous features [40,41,42,43,44,45,46]. They possess similar features, such as biocompatibility [44,47] and biodegradability [46]. Their non-toxicity has proven that they are less harmful to humans and the environment, and thus they are often selected as solid phase materials for adsorptions of various pollutants, including pharmaceutical ones [43,48,49,50]. Moreover, they are formed from environmentally friendly sources; chitosan is formed from naturally existing resources, such as the exoskeletons of anthropoids, like shellfish, crabs and prawns [51], whereas beta-cyclodextrins can be derived from enzymatic degradation of starch [46]. Great attention has been focused on the immobilization of cyclodextrins on chitosan; their combination improves the adsorption capacity of chitosan [42,44].

Recently, coupling of adsorption processes and ultrasound irradiation have gained considerable attention due to their numerous advantages [26,52,53,54,55]. These include faster chemical reactions and mass transfer as a result of acoustic cavitation with the establishment of new adsorption sites on the adsorbent surface [26,52,53,54,55]. The influences of ultrasonic irradiation on the adsorptive removals of numerous pollutants from aqueous solutions have been reported in the literature [26,52,53,54,55,56,57,58].

Therefore, the objective of the present study was to synthesise magnetic mesoporous carbon/β-cyclodextrin–chitosan (MMPC/Cyc-Chit) nanocomposite as a sorbent for the elimination of fluoroquinolones. Factors that play a role in the adsorptive removal of the fluoroquinolones by MMPC/Cyc-Chit nanocomposite were examined; namely, sonication power level, sample pH and initial concentration of DANO, LEVO and ENRO. The overall process was to utilize cheap and readily available material for nanocomposite synthesis and ultrasonic radiation for superior removal efficiency. The incorporation of biodegradable polymers such as chitosan and β-cyclodextrin to magnetic mesoporous carbon resulted in a nanocomposite with super-adsorbent activities considering high surface area and adsorption capacities. The application of MMPC/Cyc-Chit nanocomposite for removal of fluoroquinolones has been reported for the first time.

## 2. Materials and Methods

### 2.1. Materials and Reagents

Chemicals reagents used for this study were of analytical grade, and Ultra-pure water (Direct-Q^®^ 3UV-R purifier system Millipore, Merck, Darmstadt, Germany) was used throughout the duration of the experiments. Danofloxacin (99.7%) (DANO), enrofloxacin (99.0%) (ENRO), levofloxacin (99%) (LEVO), HPLC grade ethanol, methanol and acetonitrile were used, along with acetic acid, sodium hydroxide, ammonium hydroxide, ferrous chloride, ferric chloride, starch, chitosan, β-cyclodextrin and ortho-phosphoric acid purchased from Sigma-Aldrich (St. Loius, MO, USA). A synthetic sample mixture of the fluoroquinolones (FQs) stock solution was prepared by dissolving appropriate amounts of DANO, ENRO and LEVO in small amounts of methanol. The mixture was then diluted with ultra-pure water to a final volume of 100 mL. The solution were stored in to refrigerator at 4–8 °C.

### 2.2. Instrumentation

The synthesized adsorbent material was analyzed utilizing different techniques of characterization in order to determine its structural suitability for adsorption of the fluoroquinolones (DANO, ENRO LEVO). X-ray diffraction (XRD) patterns were recorded using a PANalytical X’Pert X-ray diffractometer (PANalytical BV, Almelo, Netherlands) utilizing Cu Kα radiation (λ = 0.15406 nm) in the 2θ range 4–90 at room temperature. The Fourier transform infrared (FT-IR) Perking–Elmer spectrum 100 spectrometer (Perkin-Elmer, Shelton, CT, USA) using the potassium bromide (KBr) pellet technique in a region of 4000–400 cm^−1^ was used to report the infrared spectrum for the prepared material. Surface characteristics such as porosity and area of the as-prepared material were analyzed by using the Brunauer–Emmett–Teller (BET) 77 K using an ASAP2020 porosity and surface area analyzer (Micrometrics Instrument Corp., Norcross, GA, USA).

The samples were degassed was at 100 °C for 3 h using N_2_ gas before analysis. Adjustments for pH where necessary were performed using an OHAUS starter 2100 pH meter (Pine Brook, NJ, USA). The surface charge/point of zero charge was evaluated for the as-prepared material using a Nano-ZS Zetasizer (Malvern Instruments, Malvern, UK). The pH was adjusted within the range of 2.0–11.0 by the addition of 0.1 mol L^−1^ acetic acid and ammonium solution to each solution with 37 mg of adsorbent material. A Scientech Ultrasonic cleaner (Labotec, Midrand, South Africa) with a volume of 5.7 L (internal dimensions: 300 × 153 × 150 mm) was used to facilitate the adsorption process. The ultrasonic system was equipped with a variable frequency and power setting. In this study, the frequency was fixed at 50 Hz and the emission power of 150 W. The system has 5 power levels (1 (weakest) to 5 (strongest)), this power setting is used to reduce or increase the size of the cavitation bubble implosion force. Therefore, the sonication power levels were varied. The analysis of the antibiotics was performed using an Agilent HPLC 1200 Infinity series, equipped with a photodiode array detector (Agilent Technologies, Waldbronn, Germany). Chromatograms were recorded at 290 nm. An Agilent Zorbax Eclipse Plus C18 column (3.5 μm × 150 mm × 4.6 mm) (Agilent, Newport, CA, USA) was operated at an oven temperature of 25 °C. The mobile phase (water with 10 mmol L^−1^ of phosphoric acid; the pH adjusted to 3.29 with triethylamine): acetonitrile (85.7:14.3, *v*/*v*) at a flow rate of 1.5 mL min^−1^. All chromatographic experiments were carried out 25 ± 3 °C while the injection volume was 10 L for all samples.

### 2.3. Preparation of the Nanocomposite

#### 2.3.1. Synthesis of Mesoporous Carbon (MPC)

Modified version of the hard templating method adapted from literature was used in the synthesis of mesoporous carbon [59]. Briefly, in a 100 mL beaker containing deionized water and equipped with magnetic stirrer for easy dissolving starch was used. The mixture was then heated over an oil bath at 120 °C to form a homogenous solution with continuous stirring at 200 rpm. Silica solution was added dropwise at approximately 1 drop per second using a burette with continuous stirring until the starch had completely dissolved. Thereafter, the solution was transferred onto a glass petri dish and left to cool at an ambient room temperature. A gel-like material was formed and dried at 60 °C in an oven for 1 h and further carbonized with the gentle flow of nitrogen gas at 500 °C for 3 h. Once carbonized the material was stirred for 24 h at 70 °C in a sodium hydroxide (30 wt %) solution to remove silica. The formed product was washed with a mixture of ethanol and water (1:1) and filtered under vacuum. The filtered product was then oven dried at 60 °C for 2 h.

#### 2.3.2. Preparation of Magnetic Mesoporous Carbon Coated with Chitosan and β-CD

Ferrous and ferric chloride solutions were dissolved in ultrapure water at a Fe^2+^/Fe^3+^ ratio of 1:2 and stirred for 5 min. Then 3 g of β-CD and 4 g MPC were added into the iron solution with vigorous stirring along with the addition of diluted sodium hydroxide solution (1.0 mol L^−1^) while heating at 80 °C for 1 h. That solution was then filtered by vacuum filter and washed with methanol plus water. The filtrate was then dried in an oven at 60 °C for 24 h. Chitosan flakes were modified based on a method described by [42]. Briefly, 3 g of chitosan flakes was dissolved in 50 mL of 3% acetic acid. Prepared magnetic material was then added to the solution of chitosan and this mixture was transferred to a round bottom flask. These were ultra-sonicated to facilitate dispersion were the pH of the prepared mixture was adjusted to 8.0–9.0 by means of diluted sodium hydroxide solution. Thereafter, it was filtered and washed with mixture of ethanol (50:50) plus water until the pH reached about 7, and oven dried at 40 °C.

### 2.4. Batch Adsorption Studies

Batch adsorption method was employed for adsorption studies. This was achieved by adding a specific mass of adsorbent (10–30 mg) to 25 mL synthetic sample solutions containing a mixture of FQ antibiotics (that is DANO, ENRO and LEVO) at a concentrations of 10 mg L^−1^. The pH of the synthetic sample solutions (5–9) were adjusted using 0.1 mol L^−1^ HCl and 0.1 mol L^−1^ NaOH. The adsorption process was carried out using an ultrasonic bath. The frequency of the ultrasonic bath was fixed at high 50 Hz while the sonication power level was varied between 2 (60 W or 40% of total power and 5 (150 W or 100% of total power). Once the adsorption processes was completed, the adsorbent and sample solution were separated using an external magnet. The supernatant was filtered by using 0.22 µm syringe filters and the residual FQ antibiotic concentration in the solution was determined HPLC-PDA.

A response surface methodology constructed by a central composite design (CCD) was used for the optimization of the most influential parameters for the removal of FQ antibiotics. These factors include sample pH, mass of adsorbent (MA) and sonication power level (SP). The removal efficiency (%RE) was used as an analytical response. The optimization process was carried out using Statistica version 13. When the optimal conditions were achieved, the adsorption isotherm and kinetics for the removal of FQ antibiotics were examined.

Under optimum conditions, Langmuir, Freundlich, Hill and Langmuir-Freundlich isotherm models (Table 1) were used to study the interaction between the prepared MMPC/Cyc-Chit nanocomposite and FQ antibiotic mixture. To achieve this, model solutions containing different concentrations selected FQs antibiotics mixture (5–80 mg L^−1^) were used.

The kinetic studies performed using an initial concentration of 50 mg L^−1^ were used to explain the rate and mechanism of the adsorption process. The kinetics models, such as pseudo-first-order, pseudo-second-order and intraparticle diffusion, were employed to analyze the equilibrium kinetic data. The thermodynamic studies were carried out using a concentration of 50 mg L^−1^ at different temperatures: 25, 35 and 40 °C.

### 2.5. Regeneration and Reusability (Recyclability) of the Nanocomposite

To investigate the regeneration capability of the MMPC/Cyc-Chit nanocomposite, 36 mg of adsorbent was placed into 25 mL of 10 mg L^−1^ FQ antibiotic solution. The mixture was sonicated for 30 min, and after the adsorption process had been completed, the separation of adsorbent by an external magnet was done. The adsorbent was then sonicated with a mixture of 10 mL of acidified water and acetonitrile mixture (55:45 ratio) for 10 min to remove the adsorbed FQs. The water was obtained by adjusted ultrapure deionized to pH 3 using *ortho*-phosphoric acid. It should be noted that 10 min desorption time was enough to remove all the analytes adsorbed. An external magnet was applied to facilitate the decantation of the desorption solvent. Desorption solution containing the FQs was analyzed using HPLC-PDA. After decantation, the adsorbent was washed with the desorption solvent; filtered; and finally, washed two times with ultrapure water and dried at 60 °C for 2 h. The above procedure was repeated 10 times.

### 2.6. Application in Real Water Samples

Wastewater (influent and effluent) samples were collected from a wastewater treatment plant (WWTP) in Pretoria, South Africa. River water and tap water were collected from the Apies River (Pretoria, South Africa) and the University of Johannesburg laboratory (Johannesburg, South Africa). The sample collection was performed during October 2019. The wastewater and river water samples were kept in 1 L glass amber bottles and transported to the laboratory to be stored at 4 °C before adsorption studies. The physicochemical characteristics, such as pH; conductivity; total dissolved solids (TDS); and dissolved organic carbon of wastewater, laboratory tap water and river water, are presented in summarized in Table A1. In addition, the concentrations of major elements such as calcium, magnesium, sodium and iron are presented in Table A1.

## 3. Results and Discussion

### 3.1. Characterization

#### 3.1.1. X-ray Diffraction Spectroscopy

**Figure 1:**
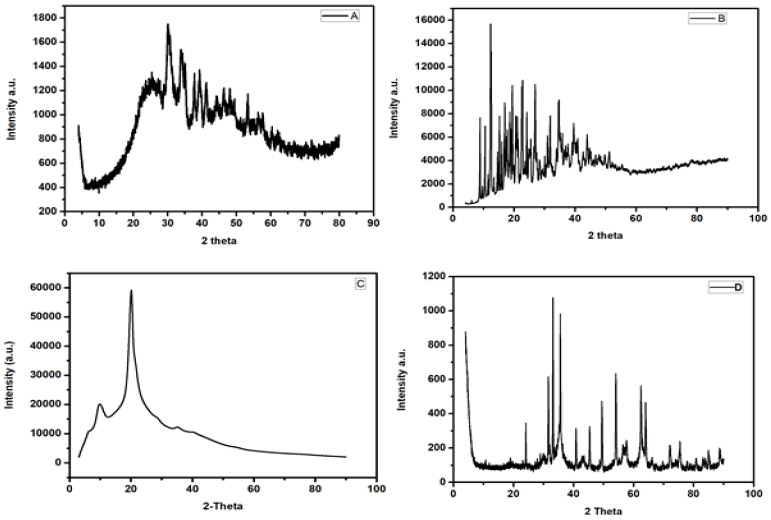
XRD of (**A**) mesoporous carbon, (**B**) beta-cyclodextrin, (**C**) chitosan and (**D**) MMPC/Cyc-Chit nanocomposite composite.

Figure 1 shows the XRD patterns of the (a) mesoporous carbon, (b) β-cyclodextrin, (c) chitosan and (d) MMPC/Cyc-Chit nanocomposite. The XRD patterns for chitosan, β-cyclodextrin and mesoporous carbon are comparable with those reported in the literature [50,59,60]. The XRD pattern for MMPC/Cyc-Chit nanocomposite shows diffraction peaks at 2θ = 31.3°, 35.7°, 42.8°, 54.1°, 56.8° and 63.2°. These diffraction peaks correspond to the magnetite planes indexed to (220), (311), (400), (422), (511) and (440). These results confirmed the importation of iron oxide nanoparticles (Fe_3_O_4_) in the nanocomposites. Moreover, they were in agreement with other results in literature [42,45].

#### 3.1.2. Fourier Transform Infrared Spectroscopy

**Figure 2:**
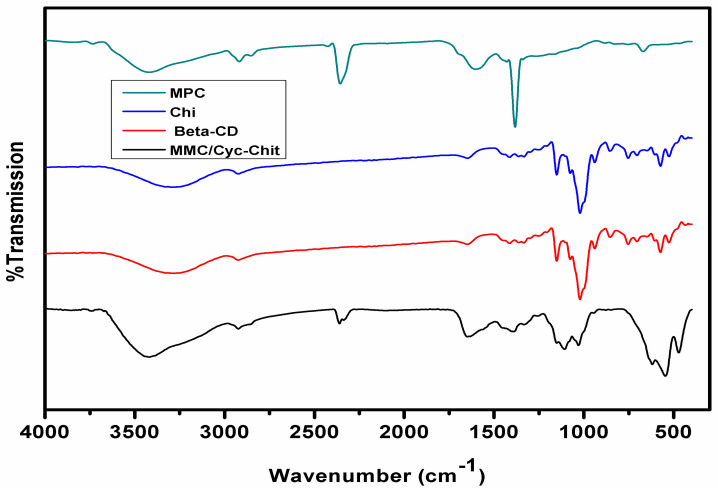
FT-IR spectra of MPC, Chi, beta-CD and MMPC/Cyc-Chit composite.

FTIR spectra for mesoporous carbon (MPC), β-cyclodextrin (β-CD), chitosan (Chi) and MMPC/Cyc-Chit nanocomposite are presented in Figure 2. The FTIR spectrum of MPC (Figure 2) reveals the peaks at 2924–2889 cm^−1^ and 1384 cm^−1^ which were ascribed to the stretching and bending of CH_3_ and CH_2_ stretching [37], whereas the broad peak at 3439 cm^−1^ was attributed to the O–H stretching. The band at 1615 cm^−1^ was assigned to the C=O vibration of carbonyl groups [39,61]. In addition, the CH_3_ stretching and unsaturated sites were observed at 2361 cm^−1^ [37]. The major bands for β-cyclodextrin and chitosan (Figure 2) were allocated as follows: 1024 cm^−1^ for (R-1, 4-bond skeleton vibration of β-CD); 1649–1656 cm^−1^ for C–N and C=O (NHCO (amide I)) stretching vibrations; and 3280–3353 cm^−1^ (O–H and N–H stretching vibrations) [42,45]. In addition, the peaks at 1586 and 1153 cm^−1^ were assigned to the N–H stretching vibration (primary amine) and antisymmetric glycosidic linkages [42]. The MMPC/Cyc-Chit nanocomposite shows two characteristic absorbance bands centered at 1652 and 1597 cm^−1^, which correspond the C=O stretching vibration of NHCO (amide I) and N–H bending of NH_2_, respectively [42].

#### 3.1.3. Nitrogen Adsorption–Desorption

The important textural properties that influence the quality and application of an adsorbent, especially for adsorptive removal of pollutants in matrices that are complex (such as wastewater and polluted river waters), are the porosity and specific surface area [24,28,36,62,63]. It has been reported that the two properties are significant because they are strongly related to the maximum adsorption capacity of the adsorbent [24,27,28,36,62,63,64]. Textural properties of the nanocomposite are presented in Table 2. The results confirmed that incorporating chitosan and β-cyclodextrin into magnetic mesoporous carbon resulted in a superabsorbent with high specific surface area (1264 m^2^ g^−1^). The micropore and mesopore surface areas of the nanocomposite in comparison with mesoporous carbon were used to analyse the textual properties of the prepared material. As seen in Table 2, the percentage of the surface comprised of mesopores was 60%, suggesting that the nanocomposite is predominantly a mesoporous material [28,34,35,36,37,39]. These characteristics validate the applicability of the nanocomposite for adsorption processes. According to the results in Table 2, it was anticipated that during the adsorption process, the investigated FQ antibiotics would percolate through pores of the adsorbents. These findings were in agreement with SEM results, and they both confirm that the prepared adsorbent possesses outstanding characteristics which endorse it for wastewater treatment using adsorption technology.

#### 3.1.4. Point of Zero Charge

**Figure 3:**
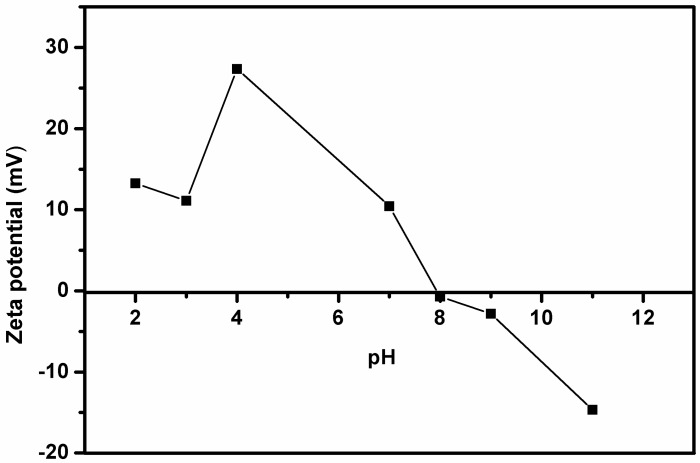
Determination of pHpzc of nanocomposite.

The pH of the FQs solution might have an effect on their adsorption on the surface of the MMPC/Cyc-Chit nanocomposite. Moreover, the pH of the sample solution was used to assess the distribution percentage of the investigated FQ species during their adsorption process. For example, subject to the pH of the sample solution, the surface of the nanocomposite could be protonated or deprotonated, thereby changing the surface charge of an adsorbent. Therefore, it is important to investigate the pH at which negative and positive charges are equal, also known as pH at point of zero charge (pH_pzc_). This point will as assist in the determination of the possible adsorption mechanism. Therefore, the influence of pH onto the zeta potential of MMPC/Cyc-Chit nanocomposite was evaluated and results are shown in Figure 3. The surface MMPC/Cyc-Chit nanocomposite was positively charged at pH values lower than 8 and the pH_pzc_ value was estimated as 8.0. This implied that MMPC/Cyc-Chit nanocomposite has a negative charge above pH = 8.0.

### 3.2. Optimization

The batch adsorption process was optimized using the RSM-ased CCD, and the design matrix together with respective responses obtained at the equivalent experimental conditions are indicated in Table 3. Statistica software was used to generate second-order polynomial model which used to explain the adsorption process of FQ antibiotics onto the MMPC/Cyc-Chit nanocomposite. The removal efficiency was used as the dependent variable or analytical response. The *R*^2^ values were used to assess the performance of the RSM model, and they were found to be 0.9985, 0.99876 and 0.9975 for DANO, ENRO and LEVO, respectively. These findings revealed the best agreement between the actual and predicted responses. Moreover, these results proposed that about 99% of the total variation in removal efficiency was attributable to the experimental factors.

**Figure 4:**
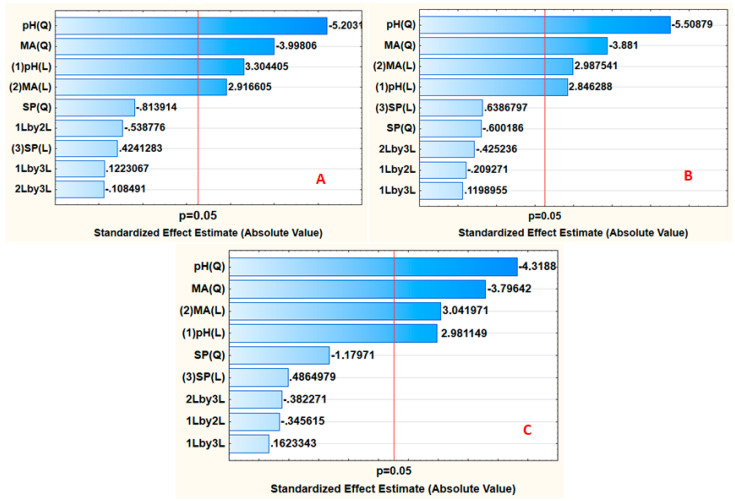
Pareto chart of standardized effects for adsorption of (**A**) DANO, (**B**) ENRO and (**C**) LEVO). MA = mass of adsorbent; SP = sonication power; 1Lby2L shows the interaction between pH and MA; 2Lby3L shows the interaction between MA and SP; 1Lby3L shows the interaction between pH and SP.

The validity and appropriateness of the RSM model, as well as the estimation of the most significant independent variables and their interactions, were examined by analysis of variance (ANOVA). The ANOVA results are reproduced in the form of Pareto charts (Figure 4). The importance of an independent variable was evaluated by the magnitude of the bar length. If the length of the bar passes the red line (0.05 confidence level line), this phenomenon suggests that the corresponding independent factor is significant at a 95% confidence level. As seen in Figure 4, the mass of the adsorbent and sample pH were significant at the 95% confidence level for every sample investigated. This implied that they had more influence on the analytical response.

#### 3.2.1. Response Surface Methodology

**Figure 5:**
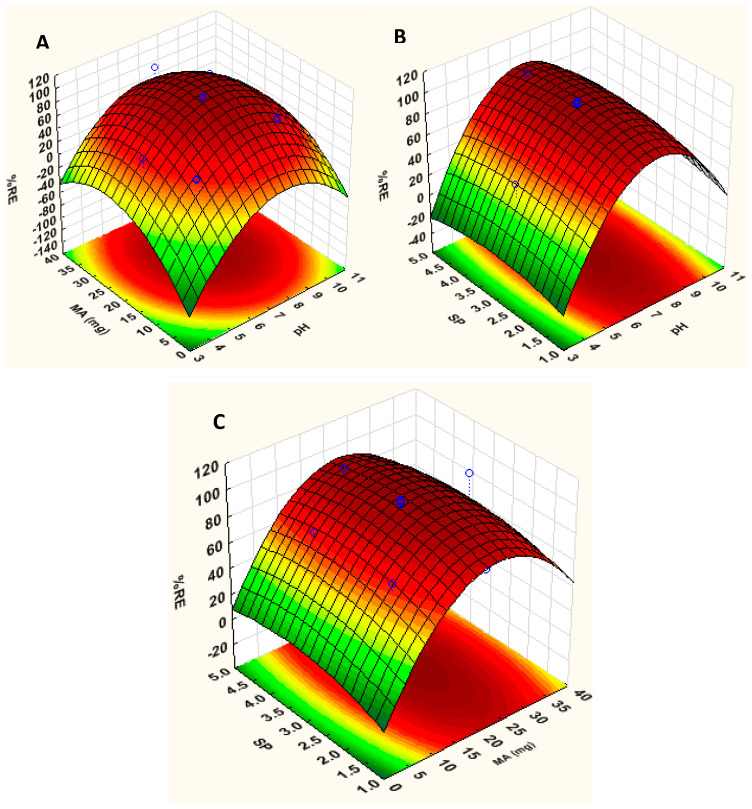
The 3D surface response plots describing the interactions of the parameters investigated. (**A**) interaction between sample pH and mass of adsorbent (MA); (**B**) interactions between sonication power (SP) and sample pH and (**C**) interactions between SP and MA.

**Figure A1:**
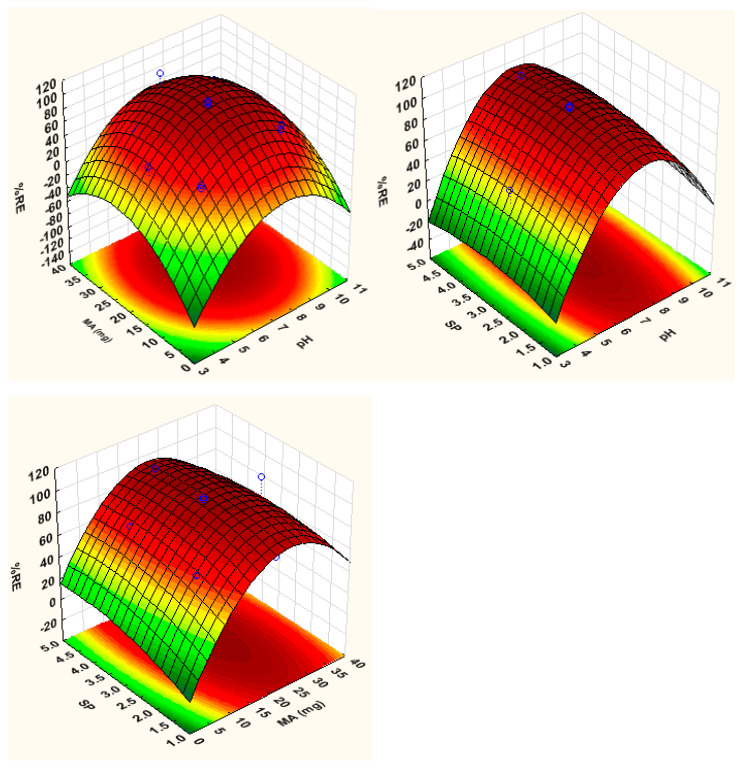
The 3D surface response plots describing the interactions of investigated parameters (ENRO).

**Figure A2:**
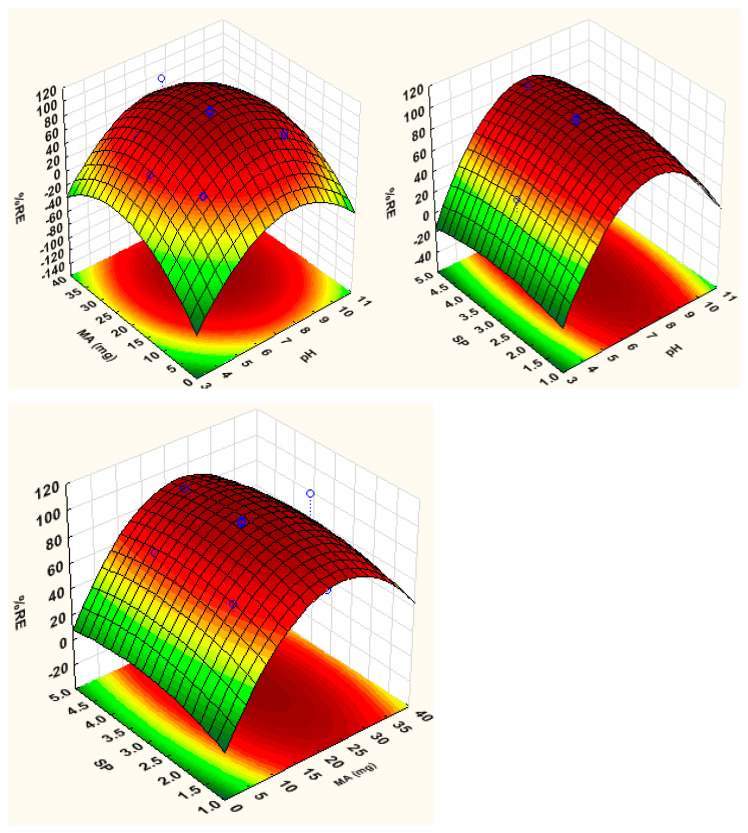
The 3D surface response plots describing the interactions of investigated parameters (LEVO).

Three-dimensional (3D) response surface plots were constructed to investigate the effect of each variable on the removal efficiency, and their interactions (Figure 5, Figure A1 and Figure A2). The effects of sample pH, mass of adsorbent (MA) and sonication power level (SP) were concurrently examined for the adsorptive removal of FQs from synthetic samples. Figure 5A shows the 3D plot of sample pH versus mass of adsorbent. As seen in Figure 5, both mass of adsorbent and the sample pH played a critical role in removal of FQ antibiotics from aqueous solutions. This might be because sample pH affects the ionization of analytes and the charge on surface of adsorbent. Based on Figure 5A,B, the removal efficiency increased with increasing sample pH, and the maximum removal was achieved between pH 6 and 8. Below and above these values, a decrease in analytical response was observed. This is because DANO, ENDRO and LEVO can exist in three forms in aqueous systems, that is, cationic (pH > pK_a2_), zwitterionic (pKa1 ≤ pH ≤ pK_a2_) and anionic (pH < pK_a1_), and these forms are pH-dependent [65,66,67,68]. Consequently, the adsorption mechanism is also dependent on the adsorbent surface charge. For instance, the FQ antibiotics can be adsorbed by a negatively or positively charged adsorbent using cation exchange through protonation of amine group or electrostatic interaction due to the deprotonation of carboxylic groups [39,61,65,66,67,69,70,71,72].

At lower pH values, the FQs are predominately in cationic forms due to a high concentration of hydronium ions [61,68,73,74]. This results in lower removal efficiencies due to the competition between the adsorbate and small molecules of hydronium ions which can fill the available active sites. Additionally, the pH_pzc_ of the material was found to be 8, indicating that the charges on surface of the nanocomposite are positive charges. Therefore, lower removal efficiencies can also be attributed to electrostatic repulsion between positively charged nanocomposite and cationic forms of FQs. As the sample pH increases, the electrostatic interaction between the adsorbate/analytes and the surface of the adsorbent occurs, resulting in higher removal efficiencies. However, at pH values > pH_pzc_ value of 8, a decline in the removal efficiency was observed. These could be attributed to electrostatic repulsion between negatively charged FQs and negatively charged nanocomposite. Several researchers in the literature have observed similar findings with respect to the adsorption behavior of FQs at low and high pH values [61,65,66,67,68,69,70,71,73,74]. The results for the effect of sonication power level are shown in Figure 5B,C; it was not significant at the 95% confidence level. However, the 3D response surface plots reveal that as the sonication power levels increases, the removal efficiency also increases. As seen in Figure 5B,C, %RE values above 80% were obtained when the sonication power was 3 (90 W or 60% of the total power) and above. The increased removal efficiency can be attributed to the increase in adsorbate–adsorbent interactions due to turbulence produced by implosion of the cavitation bubbles.

#### 3.2.2. Desirability Function

**Figure 6:**
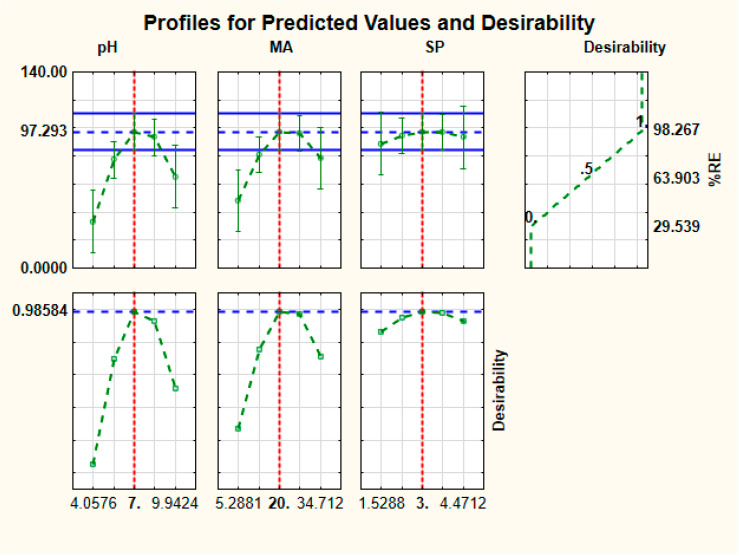
Profiles for predicated values and desirability function for removal of fluoroquinolones.

**Figure A3:**
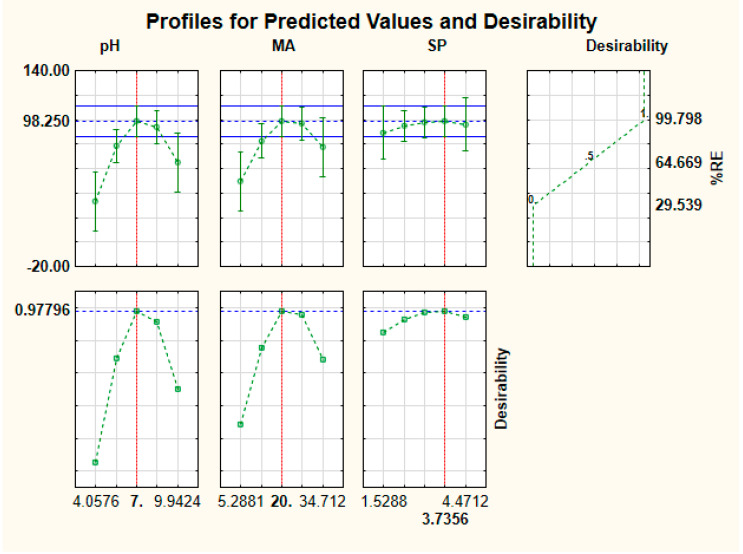
Profiles for predicated values and desirability function for removal of ENRO.

**Figure A4:**
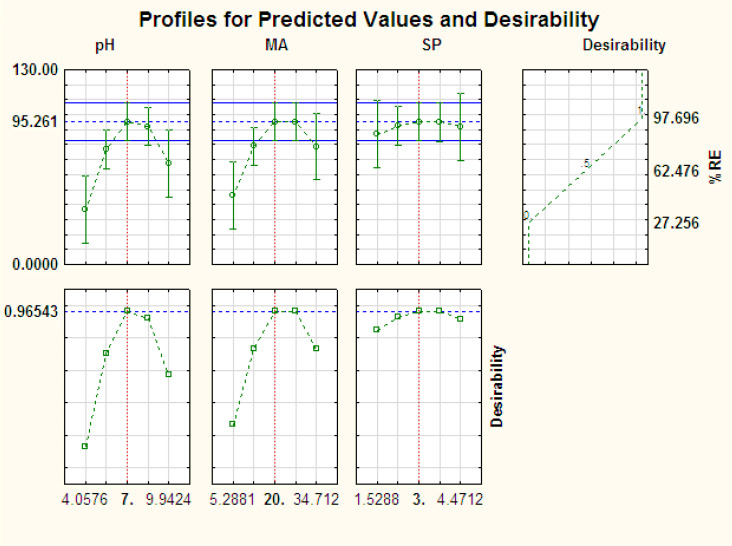
Profiles for predicated values and desirability function for removal of LEVO.

The desirability profile was used to estimate the optimum experimental conditions obtained using RSM optimization approach (Figure 6, Figure A3 and Figure A4). The optimal conditions for the removal of fluoroquinolones were sample pH: 7.0, mass of adsorbent: 36 mg and sonication power level 3. The sonication or contact time, initial concentration and sonication frequency were fixed at 30 min, 10 mg L^−1^ and 50 Hz. Under the abovementioned conditions, the predicted removal efficiencies of the model for the adsorption of DANO, ENRO and LEVO were 97.2%, 98.3 and 95.3%, respectively. To certify the acceptability of the RSM model and to confirm the agreement between the predicted and experimental removal efficiency, six replicates were carried out at the abovementioned conditions. The obtained experimental results showed removal efficiencies of 98.7 ± 1.3%, 99.1 ± 0.9% and 96.8 ± 1.2% for DANO, ENRO and LEVO, respectively. These results showed that the RSM model could be considered an accurate and valid procedure for the optimization of the adsorption process.

### 3.3. Adsorption Kinetics

**Figure 7:**
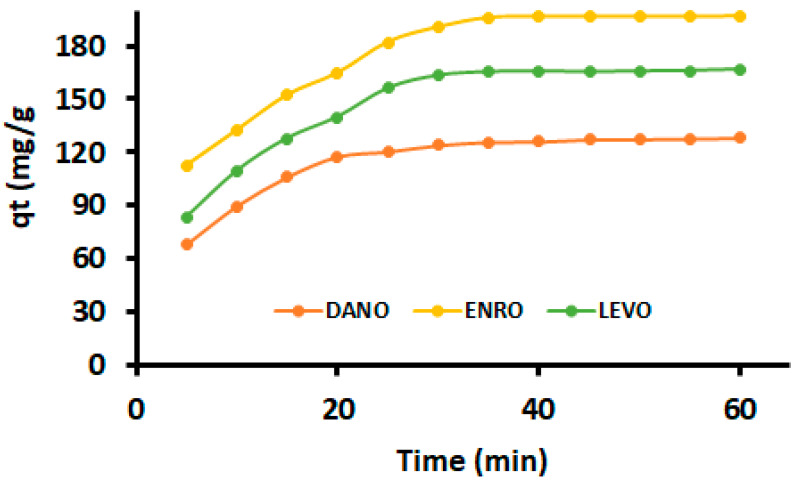
Effects of contact time on DANO, ENRO and LEVO by MMPC/Cyc-Chit kinetic modelling.

The adsorption kinetics data (Figure 7) were used to study the adsorption process of FQ antibiotics onto the surface of the nanocomposite. The data were analysed using three commonly used kinetic models; namely, pseudo-first-order, pseudo-second-order and intraparticle diffusion. The equations of these kinetic models are widely reported (See Table 1), and they were adapted from the literature [66,67,69].

The estimated parameters are presented in Table 4. As it is indicated, the R^2^ values achieved for pseudo-second-order were constantly higher compared to those of pseudo-first-order. In addition, the adsorption capacities obtained using the pseudo-second-order kinetic model were in agreement with experimental values. These outcomes suggested that the rate-determining step might be dominated by chemical interactions of FQ antibiotics with the homogenous surface of the adsorbent. The chemisorption mechanism might be driven by electrostatic attraction between the adsorbent and FQs. The dissociated forms FQ antibiotics have carboxylate and nitrogen functional groups that can bind on the positive or negative adsorbent surface.

**Figure A5:**
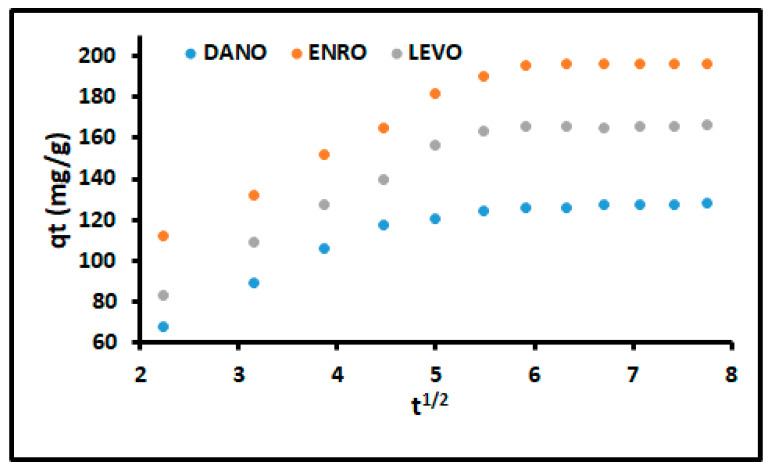
Intraparticle diffusion model.

To further understand the adsorption mechanism and the rate-controlling step, the adsorption data were fitted to the intraparticle diffusion model [74]. The plots of q_t_ versus *t*^1/2^ for the investigated adsorbates showed multi-linearity (Figure A5). These plots indicated that there were two adsorption steps that took take place. According to the literature, the steeper first-step is due to diffusion of FQ antibiotics through the solution to the mesoporous nanososorbent. The second stage is attributed to transfer of the DANO, ENRO and LEVO charged molecules into intraparticle active sites or pores of the nanocomposite. Furthermore, it was noticed that the linear part of the first step did not pass through the origin. This signified that intraparticle diffusion was not the only rate-determining step [74,75]. Therefore, it can be concluded that adsorption processes were driven by both surface adsorption and intra-particle diffusion. The intraparticle diffusion rate constants for the first and second stages (*k*_id1_, *k*_id2_), correlation coefficients and intercept, C are indicated in Table 4. The *R*^2^ values suggested that the adsorption of FQs on MMPC/Cyc-Chit nanocomposite may be dominated by intra-particle diffusion.

### 3.4. Adsorption Isotherms

**Figure 8:**
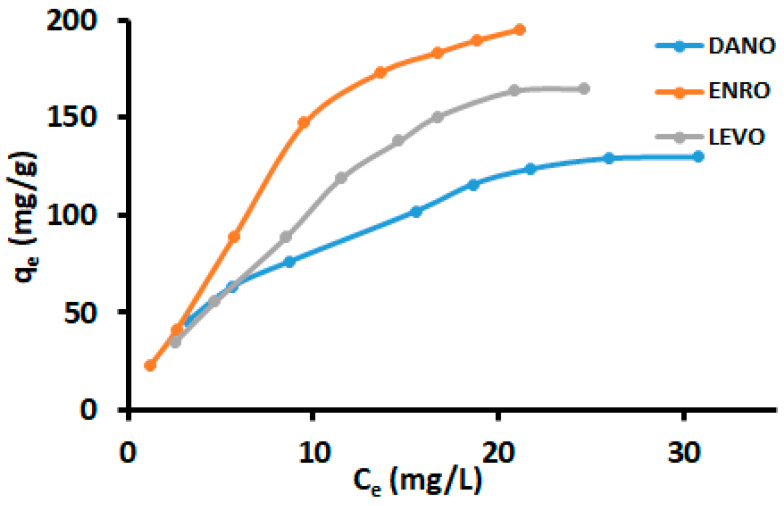
Sorption isotherms—modeling with Langmuir, Freundlich, Sips and Hill (MA: 34 mg; sonication time: 25 min; pH 7; temperature: 25 ± 3 °C).

To study the relationship between the concentration of FQs retained by the surface of the adsorbent and that of residual FQs in the bulk solution, the equilibrium studies were performed. The adsorption data were determined using Langmuir, Freundlich, Hill and Langmuir–Freundlich (Sips) isotherm models, and the model expressions are summarized in Table 1. The adsorption isotherms of FQs using the nanocomposite were carried out at 25 °C, and the pH of the solution, mass of adsorbent and contact time were set at 7, 30.0 mg and 30 min, respectively. Figure 8 demonstrates the adsorption isotherms of FQs onto nanocomposite from aqueous solutions. The isotherm models were used to derive various parameters related to the adsorption process.

Table 5 shows the summary of parameters derived from Langmuir, Freundlich, Hill and Langmuir–Freundlich (Sips) isotherm model plots. Comparing the correlation coefficients (R^2^) values for the Langmuir and Freundlich isotherm models, it was detected that DANO, ENRO and LEVO were better suited to the Langmuir model. These findings demonstrated that the adsorption process took place as a monolayer of FQs on the surface of the adsorbent. The maximum DANO, ENRO and LEVO adsorption capacities for the adsorbent were 130, 196 and 194 mg g^−1^, respectively. As seen in Table 5, the Freundlich isotherm model was also used to some extent; however, it was not as good as the Langmuir isotherm model.

The separation factor (*R*_L_) values for each adsorbate (Table 5) were used to examine wherever the adsorption process was favourable. The values were calculated from the Langmuir isotherm and they suggested that the selected FQ antibiotics were easily adsorbed onto the nanocomposite because *R*_L_ values were less than 1. In addition, the observation was also made that *R*_L_ values decrease with an increase in the initial concentration, stipulating that the adsorption of FQs was more favourable at high concentrations [76]. The equilibrium adsorption data were also modelled using three-parameter isotherms expressions (Hill and Langmuir–Freundlich isotherm models) and parameter values are illustrated in Table 5. As seen, both models confirmed that the adsorption process assumes the homogeneous monolayer on the heterogeneous surface of the nanocomposite. In addition, the Hill model exponent *n*_H_ values for DANO, ENRO and LEVO were greater than 1, indicating that the binding interaction between FQ antibiotics and nanocomposite was in the form of positive cooperativity [72].

### 3.5. Adsorption Thermodynamics

The effect of temperature in the removal of DANO, ENRO and LEVO using the nanocomposite was investigated. The thermodynamic parameters, such as Gibs energy (Δ*G*°) enthalpy (Δ*H*°) and entropy (Δ*S*°) are presented in Table 6. The values of Δ*G*° were calculated using Equation (4), whereas the Δ*H*° and Δ*S*° values were estimated from the slopes and intercepts of the plots that were obtained using Equation (4). As seen, the Δ*G*° values were negative at all investigated temperatures. This phenomenon suggested that the adsorption was spontaneous [65,66,77]. Furthermore, the positive values of Δ*H*° demonstrated that the adsorption interaction between the antibiotics and the nanocomposite was characterised by endothermic nature [24,65,66,77]. The values of Δ*H*° were higher than 20.9 kJ/mol, confirming that the adsorption processes of FQ antibiotics were dominated by a chemisorption mechanism [78]. Moreover, the positive values of Δ*S*° suggested that there is an increase in randomness at the boundary of solid/liquid phases, which might reveal the possible structural variations of the analyte and adsorbent [65,66,77].

### 3.6. Comparison of Sorption Capacities for Various Adsorbents

To compare the performance of the nanocomposite for the adsorption of FQ antibiotics, adsorption capacities of DANO, ENRO and LEVO on various adsorbents is presented in Table 7. As observed in Table 7, the adsorption capacity of the nanocomposite was comparable even better than other adsorbents reported elsewhere [25,61,65,67,69,70,71,74]. However, the adsorption capacity was lower than those reported by references [66,73].

### 3.7. Regeneration and Reusability Studies

**Figure 9:**
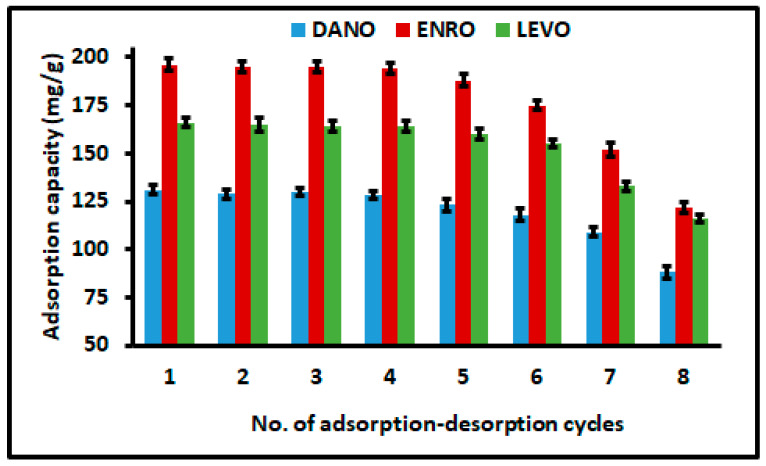
Regeneration of MMC/Cyc-Chit nanocomposite for eight successive adsorption–desorption cycles. Experimental conditions: *C*_0_ = 50 mg L^−1^; extraction time = 180 min; pH = 3.0; mass adsorbent = 36 mg; desorption solvent: acidified water and acetonitrile mixture (55:45 ratio); desorption time = 10 min.

Regeneration and reusability for spent adsorbent are two of most crucial factors from the cost-effective point of view. This study investigated the possibility of regenerating and reusing the spent nanocomposites-loaded with FQ antibiotics. The regeneration and reusability process was performed according to the procedure described in Section 2.5. As seen in Figure 9, the regenerated nanocomposite retained 90–100% of its adsorption capacity toward the removal of DANO, ENRO and LEVO, after five cycles of the desorption–adsorption. Furthermore, the adsorption capacities of the spent adsorbent for removal of DANO, ENRO and LEVO remained at 88, 122 and 116 mg g^−1^, respectively, after the eighth cycle. Furthermore, the spent adsorbent after the eighth cycle was used for the removal of FQ antibiotics. It was observed that even though the adsorption capacities decreased, the removal efficiency remained above 95%. These results demonstrated that the nanocomposite can be reused several times without affecting its removal efficiency. Additionally, it was then concluded that the prepared nanocomposite had relatively high chemical and thermal stability.

### 3.8. Application to Real Samples

The applicability of the synthesized nanocomposite was assessed for the adsorptive removal DANO, ENRO and LEVO from real water samples; i.e., tap water, river water, influent and effluent wastewater. The river water, influent and wastewater samples were filtered using a 0.22 µm syringe filter. The target analytes were detected in influent and effluent wastewater and their concentrations ranged from 58 to 1230 µg L^−1^, whereas only traces of ENRO were detected in river water samples (Table 8). As seen, the overall removal efficiencies of DANO, DANO and LEVO in spiked water samples ranged from 90–99% and the concentration of the target analyte reduced significantly. These outcomes demonstrate the good performance of the adsorbent for water and wastewater treatment.

### 3.9. Cost Analysis for the Preparation of Adsorbent

The cost of the adsorption process is predominantly dependent on the cost of adsorbent used for the removal of organic and inorganic pollutants from wastewater [79]. Therefore, relatively low-cost materials with properties that are comparable to commercially available adsorbents are required. The cost estimation breakdown for the preparation of the mesoporous carbon and nanocomposite is presented in Table 9. In comparison with the other commercially available nanomaterials, such as multi-walled carbon nanotubes (R2354/g, Sigma-Aldrich), graphene oxide (R2163/g, Sigma-Aldrich) and mesoporous carbon (R2623/5 g, Sigma-Aldrich), the cost of mesoporous carbon and nanocomposite is much cheaper. A kilogram of the prepared nanocomposite will cost about R23262.50 ($1324.85). The regeneration and reusability studies of the nanocomposite further reduce the cost of the adsorbent, since one batch can be reused at least five times. This confirms that the production of the MMPC/Cyc-Chit nanocomposite is economical and sustainable. Furthermore, regeneration and reusability are value-added properties of MMPC/Cyc-Chit nanocomposite as a promising adsorbent in the treatment of wastewater contaminated with emerging contaminants. The incorporation of magnetic nanoparticles led to the easy and fast separation (using external magnet) of adsorbent from aqueous solutions. The spent adsorbent can be first treated by the Fenton process (advanced oxidation processes, AOPs) before degrading the adsorbed pollutants. Furthermore, chitosan and β-cyclodextrin are types of fully biodegradable natural materials. This means that once the organic pollutants have been degraded by Fenton process, the adsorbent can be buried in the soil to allow biodegradation process.

## 4. Conclusions

A magnetic mesoporous carbon/β-cyclodextrin–chitosan (MMPC/Cyc-Chit) hybrid nanocomposite adsorbent was synthesized by the facile hydrothermal method. The prepared MMPC/Cyc-Chit adsorbent was characterized using BET, XRD, TEM and FTIR. The adsorption capabilities of the synthesized nanocomposite were studied in a multicomponent system employing the ultrasound-aided removal process. The effects of independent variables (sample pH, mass of adsorbent and sonication power level) were investigated and optimized using RSM based on the CCD. The use of the ultrasound system led to rapid achievement of equilibrium and improved the adsorption process due to intensified mass transfer as well as the enhanced affinity between adsorbate and adsorbent due to acoustic cavitation effects. The adsorption isotherm equilibrium data followed the Langmuir model, suggesting that the surface of the adsorbent is coated as monolayer coverage by DANO, ENRO and LEVO molecules. Furthermore, the three-parameter models confirmed that the adsorption process assumes the homogeneous monolayer on the heterogeneous surface of the MMPC/Cyc-Chit nanocomposite. The kinetic data were best described by the pseudo-second-order model proposing that the adsorptive removal process was dominated by chemisorption. The thermodynamic parameters which include ΔG°, ΔH°, and ΔS° indicated the adsorption process was feasible, spontaneous and endothermic in nature. In addition, the magnitude of ΔH° suggested that the removal of FQ antibiotics was via chemisorption and these findings agreed with the kinetic data. The synthesized MMPC/Cyc-Chit nanocomposite showed relatively high chemical and thermal stability and reusability over five adsorption–desorption cycles. The adsorption process was also applied in the removal of fluoroquinolones from real wastewater, tap water and river water samples. The results obtained demonstrated that MMPC/Cyc-Chit nanocomposite can be applied in water and wastewater treatment process.

## Figures and Tables

**Table 1:** Adsorption isotherms and kinetics models equations.

Isotherm Models	Isotherm Expression	Definition of Terms
Langmuir	$\frac{C_{e}}{q_{e}}=\frac{1}{q_{\max}K_{L}}+\frac{C_{e}}{q_{\max}}$ $R_{L}=\frac{1}{1+K_{L}C_{0}}$	*q*_max_: theoretical monolayer adsorption capacity (mg g^−1^) *C*_e_: equilibrium concentration (mg L^−1^), *q*_e_: the amount of adsorbate adsorbed per unit weight of adsorbent (mg g^−1^) *C*_0_: initial concentration (mg g^−1^) *K*_L_: Langmuir equilibrium constant (L mg^−1^) *R*_L_: separation factor
Freundlich	$\ln q_{e}=\ln K_{F}+\ln C_{e}$	*K*_F_: Freundlich constant (L g^−1^) *n*: is the Freundlich exponent (g L^−1^)
Hill	$q_{e}=q_{H}\frac{C_{e}^{n_{H}}}{K_{D}+C_{e}^{n_{H}}}$	*n*_H_ and *K*_D_: Hill isotherm constants *q*_H_: maximum equilibrium adsorption capacity (mg/g).
Langmuir–Freundlich	$\frac{1}{q_{e}}=\frac{1}{Q_{\max}K_{s}}{\left(\frac{1}{C_{e}}\right)}^{\frac{1}{n}}+\frac{1}{Q_{\max}}$	*K*_S_: Sips equilibrium constant (1/mg) *Q*_max_: maximum adsorption capacity (mg g^−1^) *n*: surface heterogeneity
Pseudo-first order	$\ln\left(q_{e}-q_{t}\right)=\ln q_{e}-k_{i}t$	*K*_1_: rate constant (min^−1^) *q*–*q*_e_: amount of absorbate at equilibrium (mg g^−1^)
Pseudo-second order	$\frac{1}{q_{t}}=\frac{1}{k_{2}\;q_{e}{}^{2}}+\frac{1}{q_{e}}t$	*K*_2_: Equilibrium rate constant (g mg^−1^ min^−1^) *q*–*q*_e_: amount of adsorbent at equilibrium (mg g^−1^)
Intra-particle diffusion	$Q_{t}=k_{i}t^{\frac{1}{2}}+C$	*Q*_t_: amount of solute on surface of sorbent at time t (mg g^−1^) *K*_i_: intraparticle diffusion constant (mg g^−1^ min^1/2^)

**Table 2:** Characteristics of adsorbent material; BET surface area; pore volume parameters of MPC and MMPC/Cyc-Chit.

Surface Properties	Mesoporous Carbon	Nanocomposite
S_BET (_m^2^ g^−1^)	1181	1264
Total pore volume (cm^3^ g^−1^)	2.54	4.65
Average pore size (nm)	7.93	8.61
t-Plot S_mesopore_ (m^2^ g^−1^)	728	755
t-Plot S_micropore_ (m^2^ g^−1^)	453	509

**Table 3:** The design matrix and the results of the two-level fractional factorial design.

Run	pH	MA	SP	DANO	ENRO	LEVO
**1**	5	10	2	33.7	33.7	36.6
**2**	5	10	4	36.7	39.5	37.5
**3**	5	30	2	49.1	49.1	51.4
**4**	5	30	4	45.3	45.3	46.0
**5**	9	10	2	70.8	65.0	66.8
**6**	9	10	4	71.0	71.0	71.8
**7**	9	30	2	71.9	74.7	76.0
**8**	9	30	4	75.1	75.1	72.7
**9**	4.1	20	3	45.4	45.4	47.3
**10**	9.9	20	3	59.1	59.1	66.9
**11**	7	5.3	3	29.5	29.5	27.3
**12**	7	35	3	96.2	96.2	97.7
**13**	7	20	1.5	85.4	85.4	81.5
**14**	7	20	4.5	96.2	99.1	97.6
**15 (C)**	7	20	3	97.9	97.9	94.8
**16 (C)**	7	20	3	97.8	97.8	95.2
**17 (C)**	7	20	3	96.9	99.8	96.2
**18 (C)**	7	20	3	98.3	98.3	94.9
**19 (C)**	7	20	3	96.1	96.1	95.0

**Table 4:** Parameters for the various kinetic models fitted onto data obtained for adsorbate solutions and results.

Kinetic Models	Parameters	DANO	ENRO	LEVO
	*q*_expt_	130	195	165
**Pseudo-first order**	*q*_e_	93.2	161	128
	*k*_1_	0.0636	0.11	0.099
	*R* ^2^	0.9400	0.9201	0.9372
**Pseudo-second order**	*q*_e_	130	196	167
	*k*_2_	0.0018	0.0011	0.0011
	*R* ^2^	0.9986	0.9971	0.9967
**Intraparticle diffusion**	*k*_id1_	17.7	24.6	24.8
	*C*_1_	32.8	56.3	29.7
	*R* ^2^_1_	0.9843	0.9866	0.9754
	*k*_id2_	1.31	0.348	0.613
	*C*_2_	118	194	162

**Table 5:** Isothermal parameters of DANO, ENRO and LEVO on MMPC/Cyc-Chit.

Two Parameter Models				Three Parameter Models			
**Langmuir isotherm**				**Hill isotherm**			
**Parameters**	DANO	ENDRO	LEVO	**Parameters**	DANO	ENRO	LEVO
***q*_max_ (mg g^−1^)**	130	196	165	***q*_H_**	129	195	164
***K*_L_ (L g^−1^)**	0.14	0.24	0.086	***n*_H_**	2.7	2.3	2.1
***R*_L_**	0.13–0.42	0.087–0.22	0.11–0.54	***K*_d_**	115	27.6	84.9
***R*^2^**	0.9911	0.9950	0.9934	***R*^2^**	0.9946	0.9859	0.9858
**Freundlich isotherm**				**Langmuir-Freundlich (Sips) isotherm**			
***K*_F_**	27.2	20.7	18.2	***Q*max**	135	208	170
***n***	2.1	1.3	1.4	***n***	1.1	1.1	1.3
***R*^2^**	0.9884	0.9810	0.9806	***K*_s_**	0.097	0.065	0.050
				***R*^2^**	0.9902	0.9884	0.9878

**Table 6:** Thermodynamic parameters for DANO, ENRO and LEVO sorption on MMPC/Cyc-Chit.

Analytes	*T* (K)	Δ*G* (kJ mol^−1^)	Δ*H* (kJ mol^−1^)	Δ*S* (J mol K^−1^)
**DANO**	298	−13.57	59.4	55.3
	308	−13.69		
	313	−13.74		
**ENRO**	298	−15.52	61.8	96.4
	308	−15.71		
	313	−15.81		
**LEVO**	298	−14.70	71.1	104
	308	−14.87		
	313	−14.94		

**Table 7:** Comparison of sorption capacities for DANO, ENRO and LEVO fluoroquinolones with various composite sorbents at 25 ± 1 °C.

Adsorbent	Adsorbate	Adsorption Capacity (mg/g)	Refs
Alkalized biochar	ENRO	40.91	[71]
Magnetic biochar-based manganese oxide composite	ENRO	7.19	[74]
iron-pillared montmorillonite	LEVO	48.61	[67]
MIL-100(Fe)	LEVO	87.34	[61]
Chitosan derived granular hydrogel with 3D structure	ENRO	388	[73]
Tb/Eu-Loaded Garlic Peels	ENRO	769	[66]
Co-modified MCM-41	LEVO	108	[65]
Fe_3_O_4_ and Fe_3_O_4_@SiO_2_	LEVO	6.85	[69]
NBent-NTiO_2_-Chit.	LEVO	90.91	[70]
Activated carbon-decorated polyacrylonitrile nanofibers	DANO, ENRO	99, 112	[25]
**MMPC/Cyc-Chit**	DANO, ENRO, LEVO	130, 196, 165	This work

**Table 8:** Adsorptive removal of fluoroquinolones in wastewater, river water and tap water samples.

Samples	Added (mg L^−1^)	DANO		ENRO		LEVO	
		Found (mg L^−1^)	%RE	Found (mg L^−1^)	%RE	Found (mg L^−1^)	%RE
**Tap water**	0	ND	-	ND	-	ND	-
	2.0	2.13 ± 0.11	99.5	1.96 ± 0.08	99.7	2.03±0.09	99.5
	5.0	5.03 ± 0.09	98.9	4.98 ± 0.05	99.0	5.11±0.02	98.9
**River water**	0	ND	-	0.098 ± 0.009	-	ND	-
	2.0	1.97 ± 0.12	97.1	2.11 ± 0.08	98.3	2.04 ± 0.02	97.7
	5.0	5.11 ± 0.09	96.8	4.95 ± 0.11	97.9	5.08 ± 0.04	95.7
**Influent**	0	0.148 ± 0.012	-	1.23 ± 0.07	-	0.573 ± 0.007	-
	2.0	2.15 ± 0.09	97.2	3.32 ± 0.04	96.3	2.58 ± 0.06	95.4
	5.0	5.21 ± 0.07	94.7	6.29 ± 0.06	90.7	5.60 ± 0.06	93.6
**Effluent**	0	0.058 ± 0.011	-	0.443 ± 0.010	-	0.078 ± 0.011	-
	2.0	2.11 ± 0.05	98.7	2.40 ± 0.06	99.0	2.13 ± 0.07	98.3
	5.0	5.07 ± 0.07	95.7	5.51 ± 0.04	98.7	5.06 ± 0.09	97.9

**Table 9:** Cost estimation breakdown for the production of magnetic mesoporous carbon/β-cyclodextrin–chitosan (MMPC/Cyc-Chit) nanocomposite.

Processes	Cost Breakdown	Temperature/Time/Mass/Volume	Unit cost (R)	Power Rating (kWh)	Price (R)
**Preparation of MPC**	Starch	15 g	1917 (2 kg)		14.37
	Sodium hydroxide	3 g	844 (1 Kg)		2.53
	Silica	20 g	1779 (500 g)		71.16
	Heating	120 °C (power = 205 W), 30 min	71.65	0.06	4.30
	Carbonization	500 °C (power = 520W), 3 h	71.65	1.56	111.77
	Cleaning	70 °C, (power = 120 W), 24 h	71.65	2.9	207.79
	Drying	60 °C (power = 240 W), 1 + 2 h	71.65	0.72	51.56
**Subtotal for 10–13 g MPC**					463.48
**Preparation of the nanocomposite**	Ferrous chloride	1g	925 (250 g)		3.70
	Ferric chloride	2 g	936 (1 kg)		1.87
	MPC	3 g	463.48 (10–13 g)		106.96
	Chitosan	3 g	1939 (100 g)		58.17
	Beta-cyclodextrin	3 g	2769.00 (100 g)		83.07
	Acetic acid	1.5 mL	1006 (2.5 L)		0.60
**Net amount of 12 g nanocomposite**					253.77
**Overhead cost (10% of net cost)**					25.38
**Total cost**					279.15

## Appendix A

**Table A1:** Physicochemical properties of water samples.

Parameters	Tap Water	River Water	Influent	Effluent
**pH**	7.7	7.6	6.64	7.11
**Conductivity (µS/cm)**	150	338	854	513
**Total dissolved solid (TDS, mg/L)**	50.6	208	432	235
**Dissolved organic carbon (DOC, mg/L)**	1.94	15.5	20.5	12.7
**Calcium (mg/L)**	2.46	6.65	19,652	18,509
**Mg (mg/L)**	1.33	4.30	15,231	12,861
**Fe (mg/L)**	0.98	3.20	634	339
**Na (mg/L)**	2.26	6.78	23,435	16,784
**K (mg/L)**	1.08	1.54	9277	6745
